# Supplementary material for: A prospective study of asymptomatic SARS-CoV-2 infection among individuals involved in academic research under limited operations during the COVID-19 pandemic
Source: PLoS One. 2022 Apr 25;17(4):e0267353. doi: 10.1371/journal.pone.0267353 (PMC9037948; doi:10.1371/journal.pone.0267353)
Supplement: S1 Appendix — (DOCX) [file pone.0267353.s001.docx]

**S1 Appendix**. Study questionnaires

**Baseline Survey**

**A. DEMOGRAPHICS**

1. What was your sex at birth?

- Male
- Female
- Intersex

2. What is your current gender identity?

- Male/Man
- Female/Woman
- Trans Male/Trans Man
- Trans Female/Trans Woman
- Genderqueer/Gender non-conforming
- Other, please Specify: ___________________________

2. What is your race? (select all that apply)

- American Indian or Alaska Native
- Asian
- Black or African American
- Native Hawaiian or Pacific Islander
- White
- Other (specify)

3. What is your ethnicity?

- Hispanic or Latinx
- Not Hispanic or Latinx
- Other (specify)

4. What is your age in years? ________ (enter number)

5. What is your primary role in UNC on-campus research:

- Principal Investigator
- Post-doctoral investigator
- Graduate Student
- Undergraduate Student
- Lab technician
- Research Assistant
- Custodian/housekeeping
- Technical Support
- Project Coordinator
- Administrator
- Study clinician
- Research Associate
- Analyst
- Teaching faculty
- Other (specify)

6. How many individuals in each age group live with you? Enter 0 if none in age group.

1. Age 0-5:____
2. Age 6-12:____
3. Age 13-18:____
4. Age 19-29:____
5. Age 30-39:____
6. Age 40-49:____
7. Age 50-59:____
8. Age 60-69:____
9. Age 70-79:____
10. 80+ years old:____

**[If a., b., or c.]**

7. Do the children in your home attend daycare or a child care center?

- Yes
- No

8. Do the children in your home currently attend school in person (elementary, middle, high school)?

- Yes
- No

9. Have childcare duties or having to supervise schooling at home during the COVID-19 stay at home orders impacted your ability to conduct your work activities?

Yes, I am working fewer hours due to child care responsibilities

Yes, I am getting less work done due to child care responsibilities

Yes, I am being less productive in terms of papers, grants, etc. due to child care responsibilities

No, I am not being impacted by childcare responsibilities

10. What is the highest level of education you have completed?

- never attended school
- kindergarten - 8th grade
- some high school
- high school equivalency (GED)
- high school graduate
- some college
- college graduate
- graduate school

11. What is your primary or native language?

- English
- Spanish
- French
- German
- Chinese (including Mandarin)
- Vietnamese
- Arabic
- Korean
- Tagalog
- Burmese
- Other (specify)

12. What was your household income for the year of 2019?

- $0-$15,000
- $15,000 - $25,000
- $25,000-$35,000
- $35,000-$50,000
- $50,000-$75,000
- $75,000-$100,000
- $100,000-$150,000
- $150,000-$200,000
- More than $200,000

13. How do you get to work?

- Walking
- Biking
- Driving a personal vehicle or carpooling with people who live in your home
- Carpooling with people who do not live in your home
- Bus

14. Has your household income changed since March 1, 2020?

- No, there have been no changes to your household income.
- Yes there have been small changes, but you are able to meet all your needs and pay bills.
- Yes, there have been moderate changes and you made cuts, but you are able to meet basic needs and pay bills.
- Yes, there have been severe changes and you are unable to meet basic needs or pay bills.

**B. SYMPTOMS**

1. During the past two weeks, have you experienced any of the following that are not attributable to another existing health condition? Please check “yes”, “no”, or “unknown” for each.

- Fever > 100.4℉ (38℃)
- Subjective fever (felt feverish)
- Chills
- new muscle aches (myalgia)
- Runny nose (rhinorrhea)
- Sore throat
- Cough (new onset or worsening of chronic cough)
- Shortness of breath (dyspnea)
- Nausea or vomiting
- Headache
- Abdominal pain
- Diarrhea (≥3 loose/looser than normal stools/24hr period)
- Sudden loss of taste or smell

**C. EXPOSURES**

During the PAST TWO WEEKS:

1. ...have you been exposed to (been in close contact or within 6ft of) someone likely to have Coronavirus/COVID-19? (check all that apply)

- Yes, someone with positive blood test (serology, antibody test, meaning past infection with the virus)
- Yes, someone with a positive nasal swab test (test for the virus, meaning active infection with the virus)
- Yes, someone with medical diagnosis, but no test
- Yes, someone with possible symptoms, but no diagnosis a test
- No, not to my knowledge

2. ...have you been suspected of having Coronavirus/COVID-19 infection?

- Yes, a positive blood test (serology, antibody test, meaning past infection with the virus)
- Yes, a positive nasal swab test (test for the virus, meaning active infection with the virus)
- Yes, a medical diagnosis, but no test
- Yes, possible symptoms, but no diagnosis by a test
- No, not to my knowledge

3. ...has anyone in your family or household been diagnosed with Coronavirus/COVID-19? (check all that apply)

- Yes, member of household
- Yes, non-household member
- No

4. …has anyone in your office/workplace been diagnosed with Coronavirus/COVID-19 (check all that apply)

- Yes, and they have been at the office/workplace at the same time as me within the past two weeks
- Yes, and we have not been at the office/workplace at the same time within the past two weeks
- No
- I don’t know

5. ...have any of the following happened to your family members because of Coronavirus/COVID-19? (check all that apply)

- Fallen ill at home
- Hospitalized
- Put into self-isolation with symptoms
- Put into self-quarantine without symptoms (e.g., due to possible exposure)
- Lost a job or been laid off
- Reduced ability to earn money
- Passed away
- None of the above

**D. RISK FACTORS***(note: The contents of this survey are collected for research purposes and are confidential)*

1. How would you rate your overall physical health?

- Excellent
- Very good
- Good
- Fair
- Poor

2. In the past month, have you missed any scheduled appointments with any health care provider?

Yes

No

If yes,

2a. What is the MAIN reason you missed appointments with any healthcare provider in the past month?

Your clinic cancelled your appointment because of COVID-19 1

Your clinic is closed because of the COVID-19 2

You had symptoms of COVID-19, so you stayed home 3

You cancelled the appointment to avoid being around others 4

You cancelled the appointment because you did not want to be in a healthcare setting 5

You felt okay or good enough 6

You didn’t have money or insurance 7

You didn’t want to take public transportation and had no other way to get there 8

You forgot to go/just missed your appointment 9

You felt disrespected by the office or medical staff 10

Other (specify) 11

Don’t know

3. Has a health or educational professional ever told you that you had any of the following health conditions? (check all that apply)

- Chronic Lung Disease such as asthma or COPD
- Heart problems/ Cardiovascular disease
- Kidney disease
- Any immunocompromising condition, including an immune disorder, organ transplant, hematologic transplant, HIV infection, cancer treated with chemotherapy or radiation, or any immunocompromising medication
- Diabetes or high blood sugar
- Obesity (BMI equal to or over 30)

4. Do you have any form of disability? (check all that apply)

- Intellectual/cognitive disability
- Physical disability that does not limit mobility in your workplace
- Physical disability that limits mobility in your workplace
- Deafness
- Blindness
- Other (please specify)

5. Are you currently pregnant?

- Yes
- No
- Don't know

6. Do you currently smoke or use tobacco products?

- Yes
- No

**[If YES]**

6a. In the past month, on how many days did you smoke part or all of a cigarette? _____ _____ days

**[SKIP NEXT QUESTION if answer to this question is 0]**

6b. On the days that you smoked cigarettes in the past month, how many cigarettes did you smoke per day, on average?

- Less than one cigarette per day 1
- 1 cigarette per day
- 2-5 cigarettes per day
- 6-15 cigarettes per day (about ½ pack)
- 16-25 cigarettes per day (about 1 pack)
- 26-35 cigarettes per day (about 1 ½ pack)
- More than 35 cigarettes per day (about 2 packs or more)

**[If NO]**

6c. If you do not currently smoke, did you ever smoke one pack of cigarettes per day or more?

- Yes
- No

7. Are you a healthcare professional?

- No
- Yes, and I currently have face-to-face contact with patients
- Yes, but I do not currently have face-to-face contact with patients

**[If YES]**

7a. If you are a health care professional, have you worked in any of the following settings since March 2020?

- Hospital Inpatient
- Hospital Outpatient
- Clinic outside a hospital
- Nursing home or group care facility
- Home health
- School Clinic
- Other Health Care Facility

8. How many rooms (total) are in your home? _____

9. What is the square footage of your home? _____

10. Do you have internet connection in your home? (Check all that apply)

- - High speed internet
  - Dial up
  - Smart phone
  - No internet

11. Are any adults living in the home an ESSENTIAL WORKER (e.g., healthcare, delivery worker, store worker, security, building maintenance)?

Yes

No

**[If YES]**

11a. Are they a FIRST RESPONDER, HEALTHCARE PROVIDER or OTHER WORKER in a facility treating COVID-19?

- Yes
- No

12. In the last two weeks, have you done the following: (check all that apply)

- Gone inside of a grocery store or pharmacy without a mask
- Gone out to a restaurant, community pool, shopping mall or other place where you gathered with people who do not live with you
- Spent time with friends, neighbors, or relatives (who you do not live with) INSIDE, no mask on
- Spent time with friends, neighbors, or relatives (who you do not live with) OUTSIDE, no mask on
- Attended a gathering with more than 10 people, such as a protest, reunion, wedding, funeral, birthday party, concert, or religious service INSIDE, no masks on
- Attended a gathering with more than 10 people, such as a protest, reunion, wedding, funeral, birthday party, concert, or religious service OUTSIDE, no masks on
- Sought care from a hospital or health care facility
- Been placed in isolation or quarantine
- Remained in your residence at all times, except for essential activities, work, or exercise
- Had close contact (within 6 feet) with people who do not live with you without a mask on

13. In the last two weeks, has anyone in your household done the following: (check all that apply)

- Gone inside of a grocery store or pharmacy without a mask
- Gone out to a restaurant, community pool, shopping mall or other place where you gathered with people who do not live with you
- Spent time with friends, neighbors, or relatives (who you do not live with) INSIDE, no mask on
- Spent time with friends, neighbors, or relatives (who you do not live with) OUTSIDE, no mask on
- Attended a gathering with more than 10 people, such as a protest, reunion, wedding, funeral, birthday party, concert, or religious service INSIDE, no mask on
- Attended a gathering with more than 10 people, such as a protest, reunion, wedding, funeral, birthday party, concert, or religious service OUTSIDE, no mask on
- Sought care from a hospital or health care facility
- Been placed in isolation or quarantine
- Remained in your residence at all times, except for essential activities, work, or exercise
- Had close contact (within 6 feet) with people who do not live with you without a mask on

14. In the past two weeks, have you had any of the following forms of travel outside of the town where you live and town where you work? (check all that apply)

- Travel by car within the state
- Travel by bus or train within the state
- Travel by car out of the state
- Travel by bus or train out of the state
- Travel by airplane
- Travel that required use of public spaces (airports, bus stations, public bathrooms/rest stops)

**E. ADHERENCE/COMPLIANCE WITH COVID-19 GUIDELINES**

1. How would you describe your primary workplace? (select all that apply)

- Laboratory
- Office that I share with other people
- Office that only I use
- Clinical area
- My job requires me to go to multiple spaces or facilities

2. With how many other people did you interact in your primary workplace on an average day before the COVID-19 pandemic (ie prior to March 2020)

- None
- 1-3 other people
- 4-6 other people
- 7-9 other people
- 10 or more other people

3. With how many other people do you interact in your primary workplace on an average day now?

- None
- 1-3 other people
- 4-6 other people
- 7-9 other people
- 10 or more other people

4. Does your typical workday require you to go to multiple buildings or sites?

- Yes
- No

5. What building is your primary workplace? (this can be a dropdown with prepopulated buildings)

5a. If you work in another building in addition to your primary workplace, please indicate (drop down)

6. What school is your appointment in?

7. What department is your hiring department?

8. In the last 2 weeks, on average, how many days a week did you come to campus? Enter value ______

9. On the days you came to campus, on average how many hours per day were you on campus? Enter value______

8. Within the past two weeks, how often have you done the following while at work:

a. Worn a mask?

- - - Never
    - Rarely
    - Occasionally
    - Often
    - Most of the time
    - All of the time

b. Maintained physical distancing (at least 6 feet of distance) between yourself and others

- - - Never
    - Rarely
    - Occasionally
    - Often
    - Most of the time
    - All of the time

c. Only met with others in a group of 10 people or less

- - - Never
    - Rarely
    - Occasionally
    - Often
    - Most of the time
    - All of the time

d. Worked from home when able

- - - Never
    - Rarely
    - Occasionally
    - Often
    - Most of the time
    - All of the time

e. Sneezed/coughed into your elbow or sneezed/coughed into a tissue, disposed of the tissue, and washed your hands

- - - Never
    - Rarely
    - Occasionally
    - Often
    - Most of the time
    - All of the time

9. In the past two weeks, how many times per day did you do the following while at work:

a. Clean your hands with soap and water or with hand sanitizer

- - - Never
    - 1-2 times per day
    - 3-5 times per day
    - 6-8 times per day
    - More than 8 times per day

b. Cleaned or sanitized communal parts of the workplace (counters, desktops, shared equipment, etc)

- - - Never
    - 1-2 times per day
    - 3-5 times per day
    - 6-8 times per day
    - More than 8 times per day

10. Within the past two weeks, did you:

a. Stay home due to a known exposure to another person with COVID-19

- - Yes
  - No
  - Did not have a known exposure

b. Stay home due to potential symptoms of COVID-19

- - Yes
  - No
  - Did not have symptoms of COVID-19

11. In the past 2 weeks, when did you put on your mask when coming to work?

- When I left home
- When I got to campus
- Before I entered any building
- When I entered my office/lab
- Other

12. Please estimate how many of your co-workers in your work space were wearing masks in the last 2 weeks.

- Fewer than 20% (1)
- 20-49% (5)
- 50-74% (6)
- 75-99% (7)
- 100% (8)

13. In the past two weeks, How many times did someone come closer to you than 6 feet?

- 0-3 (1)
- 4-7 (2)
- 8-10 (3)
- More than 10 times (4)

14. In the past 2 weeks, how many times did you eat or drink in an indoor shared space at work while you and others in the room were not wearing masks?

0-3 (1)

- 4-7 (2)
- 8-10 (3)
- More than 10 times (4)

15. Were there times in the last 2 weeks when you did not wear a mask at work all the time?

- - Yes
  - No

**[If YES]**

15a. There were times when I did not wear a mask at work because: (check all that apply)

- - I had access to a mask and did not want to wear it
  - I had access to a mask and forgot to wear it
  - I had access to a mask and had difficulty wearing it at all times
  - I had access to a mask and did not wear it because I was concerned about how others would react
  - I did not have access to a mask
  - I did not wear a mask because I was by myself in my workspace
  - Other, please specify

16. Were there times in the last 2 weeks when you came closer than 6ft from others at work?

- - Yes
  - No

**[If YES]**

16a. . There were times when I did not stay at least 6ft apart from others at work because: (check all that apply)

- - I could stay 6ft away from others but did not want to
  - I could stay 6ft away from others but forgot to
  - I tried to stay 6ft away from others but my coworkers entered my personal space
  - My duties or responsibilities required that I come within 6ft of others
  - Other, please specify

17. Were there times in the last 2 weeks when you met with more than 10 people in a group at work?

- - Yes
  - No

**[If YES]**

17a. . There were times when I met with more than 10 people in a group at work because: (check all that apply)

- - I could have met in a smaller group or avoided the meeting but I did not want to
  - I could have met in a smaller group or avoided the meeting but I forgot to
  - I felt required or compelled to attend the meeting with more than 10 people
  - Other, specify

18. Were there times in the last 2 weeks when you did not wash or sanitizer your hands at work?

- - Yes
  - No

18a. There were times when I did not wash or sanitize my hands at work because: (check all that apply)

- - I had access to hand cleansers but did not want to clean my hands
  - I had access to hand cleansers but forgot to clean my hands
  - I did not have access to soap and water or hand sanitizer
  - Other, specify

19 Were there times in the last 2 weeks when you had symptoms that could be due to COVID-19 and came into work?

- - Yes
  - No

If YES

19a. There were times in which I had symptoms that could be due to COVID-19 and came into work because: (check all that apply)

- - I did not think there was a significant chance I had COVID-19
  - I thought I might have COVID-19 but did not think I would transmit it to others
  - I thought I might have COVID-19 and might spread it to others, but this did not concern me
  - I felt required or compelled to come to work even though I told my boss/manager I had symptoms
  - I felt required or compelled to come to work and did not feel I could tell my boss/manager about my symptoms
  - Other, specify

20. In the past two weeks, how often have you done the following while not at work:

a. Worn a mask when you were in public?

- - Never
  - Rarely
  - Occasionally
  - Often
  - Most of the time
  - All of the time

b. Maintained social distancing (at least 6 feet of distance) between yourself and others who do not live with you

- - Never
  - Rarely
  - Occasionally
  - Often
  - Most of the time
  - All of the time

c. Only met with others in a group of 10 people or less

- - Never
  - Rarely
  - Occasionally
  - Often
  - Most of the time
  - All of the time

d. Sneezed/coughed into your elbow or sneezed/coughed into a tissue, disposed of the tissue, and washed your hands

- - Never
  - Rarely
  - Occasionally
  - Often
  - Most of the time
  - All of the time

21. In the past two weeks, how many times per day did you wash or sanitize your hands while not at work?

- Never
- 1-2 times per day
- 3-5 times per day
- 6-8 times per day
- More than 8 times per day

**F. RISK PERCEPTION**

1. During the PAST TWO WEEKS, how worried have you been about:

a. ...being infected with the Coronavirus/COVID-19?

- - Not at all
  - Slightly
  - Moderately
  - Very
  - Extremely

b. … friends or family being infected with the Coronavirus/COVID-19?

- - Not at all
  - Slightly
  - Moderately
  - Very
  - Extremely

c. … your Physical health being influenced by the Coronavirus/COVID-19?

- - Not at all
  - Slightly
  - Moderately
  - Very
  - Extremely

d. … your Mental/Emotional health being influenced by Coronavirus/COVID-19?

- - Not at all
  - Slightly
  - Moderately
  - Very
  - Extremely

e. …a COVID-19 diagnosis affecting your employment, personal relationships, or social worth?

- - Not at all
  - Slightly
  - Moderately
  - Very
  - Extremely

2. How much are you reading or talking about Coronavirus/COVID-19?

- Never
- Rarely
- Occasionally
- Often
- Most of the time

3. How do you feel about being back on campus for work?

- I’m comfortable and/or happy to be back on campus for work
- I am indifferent about being back on campus for work
- I am unhappy or feel forced to return to campus for work

4. How safe do you feel working in your work space with regard to contracting COVID-19?

- Not safe at all (1)
- A little safe (2)
- Somewhat safe (3)
- Safe (4)
- Very safe (5)

5. Has the Coronavirus/COVID-19 crisis in your area led to any positive changes in your life?

- None
- Only a few
- Some

6. Over the last 2 weeks, how often have you had little interest or pleasure in doing things?

- Not at all
- Several days
- More than half the days
- Nearly every day

7. Over the last 2 weeks, how often have you felt down, depressed, or hopeless?

- Not at all
- Several days
- More than half the days
- Nearly every day

8. Over the last 2 weeks, how often have you felt nervous, anxious, or on edge?

- Not at all
- Several days
- More than half the days
- Nearly every day

9. Over the last 2 weeks, how often have you been unable to stop or control worrying?

- Not at all
- Several days
- More than half the days
- Nearly every day

**Follow-up Survey**

**Symptoms**

- During the past two weeks, have you experienced any of the following that are not attributable to another existing health condition? Please check “yes”, “no”, or “unknown” for each.
  - Fever > 100.4℉ (38℃)
  - Subjective fever (felt feverish)
  - Chills
  - new muscle aches (myalgia)
  - Runny nose (rhinorrhea)
  - Sore throat
  - Cough (new onset or worsening of chronic cough)
  - Shortness of breath (dyspnea)
  - Nausea or vomiting
  - Headache
  - Abdominal pain
  - Diarrhea (≥3 loose/looser than normal stools/24hr period)
  - Sudden loss of taste or smell

**Exposures**

- During the PAST TWO WEEKS:
  - ...have you been exposed to (been in close contact or within 6ft of) someone likely to have Coronavirus/COVID-19? (check all that apply)
    - Yes, someone with positive test
    - Yes, someone with medical diagnosis, but no test
    - Yes, someone with possible symptoms, but no diagnosis by doctor
    - No, not to my knowledge
  - ...have you been suspected of having Coronavirus/COVID-19 infection?
    - Yes, has positive test
    - Yes, medical diagnosis, but no test
    - Yes, have had some possible symptoms, but no diagnosis by doctor
    - No symptoms or signs
  - ...has anyone in your family or household been diagnosed with Coronavirus/COVID-19? (check all that apply)
    - Yes, member of household
    - Yes, non-household member
    - No
  - …has anyone in your workplace been diagnosed with Coronavirus/COVID-19 (check all that apply)
    - Yes, and they have been at the workplace at the same time as me within the past two weeks
    - Yes, and we have not been at the workplace at the same time within the past two weeks
    - No
  - ...have any of the following happened to your family members because of Coronavirus/COVID-19? (check all that apply)
    - Fallen ill at home
    - Hospitalized
    - Put into self-isolation with symptoms
    - Put into self-quarantine without symptoms (e.g., due to possible exposure)
    - Lost a job or been laid off
    - Reduced ability to earn money
    - Passed away
    - None of the above

In the last 2 weeks, on average, how many days a week did you come to campus? Enter value ______

On the days you came to campus, on average how many hours per day were you on campus? Enter value______

**Risk Factors**

- In the last two weeks, have you done the following: (check all that apply)
  - Gone to the grocery store or pharmacy
  - Gone out to a restaurant, protest, community pool, or other place where you gathered with people who do not live with you
  - Gone to a friend, neighbor, or relative’s residence (that is not your own)
  - Had visitors such as friends, neighbors or relatives at your residence
  - Attended a gathering with more than 10 people, such as a reunion, wedding, funeral, birthday party, concert, or religious service
  - Sought care from a hospital or health care facility
  - Been placed in isolation or quarantine
  - Remained in your residence at all times, except for essential activities, work, or exercise
  - Had close contact (within 6 feet) with people who do not live with you
- In the last two weeks, have any other adults in your household done the following: (check all that apply)
  - Gone to the grocery store or pharmacy
  - Gone out to a restaurant, protest, or other place where they gathered with people who do not live with you
  - Gone to a friend, neighbor, or relative’s residence (that is not your own)
  - Had visitors such as friends, neighbors or relatives at your residence
  - Attended a gathering with more than 10 people, such as a reunion, wedding, funeral, birthday party, concert, or religious service
  - Sought care from a hospital or health care facility
  - Been placed in isolation or quarantine
  - Remained in your residence at all times, except for essential activities, work, or exercise
  - Had close contact (within 6 feet) with people who do not live with you
- If you have children (under the age of 18) in your household, have they engaged in any of the following activities within the last two weeks?
  - Gone out to a playground, school, daycare, or other place where they gathered with people who do not live with you
  - Gone to a friend, neighbor, or relative’s residence (that is not your own)
  - Had visitors such as friends, neighbors or relatives at your residence
  - Attended a gathering with more than 10 people, such as a reunion, wedding, funeral, birthday party, concert, or religious service
  - Sought care from a hospital or health care facility
  - Been placed in isolation or quarantine
  - Remained in your residence at all times, except for essential activities or exercise
  - Had close contact (within 6 feet) with people who do not live with you
- In the past two weeks, have you had any of the following forms of travel outside of the town where you live and town where you work? (check all that apply)
  - Travel by car within the state
  - Travel by bus or train within the state
  - Travel by car out of the state
  - Travel by bus or train out of the state
  - Travel by airplane
  - Travel that required use of public spaces (airports, bus stations, public bathrooms/rest stops)

**Adherence/Compliance with COVID-19 Guidelines**

- With how many other people do you interact in your primary workplace on an average day in the past 2 weeks?
  - None
  - 1-3 other people
  - 4-6 other people
  - 7-9 other people
  - 10 or more other people
- Within the past two weeks, how often have you done the following while at work:
  - Worn a mask?
    - Never
    - Rarely
    - Occasionally
    - Often
    - Most of the time
    - All of the time
  - Maintained social distancing (at least 6 feet of distance) between yourself and others
    - Never
    - Rarely
    - Occasionally
    - Often
    - Most of the time
    - All of the time
  - Only met with others in a group of 10 people or less
    - Never
    - Rarely
    - Occasionally
    - Often
    - Most of the time
    - All of the time
  - Worked from home when able
    - Never
    - Rarely
    - Occasionally
    - Often
    - Most of the time
    - All of the time
  - Sneezed/coughed into your elbow or sneezed/coughed into a tissue, disposed of the tissue, and washed your hands
    - Never
    - Rarely
    - Occasionally
    - Often
    - Most of the time
    - All of the time
- In the past two weeks, how many times per day did you do the following while at work:
  - Clean your hands with soap and water or with hand sanitizer
    - Never
    - 1-2 times per day
    - 3-5 times per day
    - 6-8 times per day
    - More than 8 times per day
  - Cleaned or sanitized communal parts of the workplace (counters, desktops, shared equipment, etc)
    - Never
    - 1-2 times per day
    - 3-5 times per day
    - 6-8 times per day
    - More than 8 times per day
- Within the past two weeks, did you:
  - Stay home due to a known exposure to another person with COVID-19
    - Yes
    - No
    - Did not have a known exposure
  - Stay home due to potential symptoms of COVID-19
    - Yes
    - No
    - Did not have symptoms of COVID-19
- If there were times when you did not follow UNC COVID-19 research guidelines, why did this happen?
  - There were times when I did not wear a mask at work because: (check all that apply)
    - I had access to a mask and did not want to wear it
    - I had access to a mask and forgot to wear it
    - I had access to a mask and had difficulty wearing it at all times
    - I had access to a mask and did not wear it because I was concerned about how others would react
    - I did not have access to a mask
    - I always wore a mask when in my workplace
  - There were times when I did not stay at least 6ft apart from others at work because: (check all that apply)
    - I could stay 6ft away from others but did not want to
    - I could stay 6ft away from others but forgot to
    - I tried to stay 6ft away from others but my coworkers entered my personal space
    - My duties or responsibilities required that I come within 6ft of others
    - I always stayed at least 6ft apart from others in my workplace
  - There were times when I met with more than 10 people in a group at work because: (check all that apply)
    - I could have met in a smaller group or avoided the meeting but I did not want to
    - I could have met in a smaller group or avoided the meeting but I forgot to
    - I felt required or compelled to attend the meeting with more than 10 people
    - I never met with more than 10 people in my workplace
  - There were times when I did not wash or sanitize my hands at work because: (check all that apply)
    - I had access to hand cleansers but did not want to clean my hands
    - I had access to hand cleansers but forgot to clean my hands
    - I did not have access to soap and water or hand sanitizer
    - I always washed or sanitized my hands after using the restroom, interacting with others, or touching potentially contaminated surfaces
  - There were times in which I had symptoms that could be due to COVID-19 and came into work because: (check all that apply)
    - I did not think there was a significant chance I had COVID-19
    - I thought I might have COVID-19 but did not think I would transmit it to others
    - I thought I might have COVID-19 and might spread it to others, but this did not concern me
    - I felt required or compelled to come to work even though I told my boss/manager I had symptoms
    - I felt required or compelled to come to work and did not feel I could tell my boss/manager about my symptoms
    - I did not have symptoms that could be due to COVID-19 during this period
- In the past two weeks, how often have you done the following while not at work:
  - Worn a mask when you were in public?
    - Never
    - Rarely
    - Occasionally
    - Often
    - Most of the time
    - All of the time
  - Maintained social distancing (at least 6 feet of distance) between yourself and others who do not live with you
    - Never
    - Rarely
    - Occasionally
    - Often
    - Most of the time
    - All of the time
  - Only met with others in a group of 10 people or less
    - Never
    - Rarely
    - Occasionally
    - Often
    - Most of the time
    - All of the time
  - Sneezed/coughed into your elbow or sneezed/coughed into a tissue, disposed of the tissue, and washed your hands
    - Never
    - Rarely
    - Occasionally
    - Often
    - Most of the time
    - All of the time
- In the past two weeks, how many times per day did you wash or sanitize your hands while not at work?
  - Never
  - 1-2 times per day
  - 3-5 times per day
  - 6-8 times per day
  - More than 8 times per day

Please estimate how many of your co-workers in your workspace were wearing masks in the last 2 weeks.

- Fewer than 20% (1)
- 20-49% (5)
- 50-74% (6)
- 75-99% (7)
- 100% (8)

13. In the past two weeks, How many times did someone come closer to you than 6 feet?

- 0-3 (1)
- 4-7 (2)
- 8-10 (3)
- More than 10 times (4)

14. In the past 2 weeks, how many times did you eat or drink in an indoor shared space at work while you and others in the room were not wearing masks?

0-3 (1)

- 4-7 (2)
- 8-10 (3)
- More than 10 times (4)

How safe do you feel working in your work space with regard to contracting COVID-19?

- Not safe at all (1)
- A little safe (2)
- Somewhat safe (3)
- Safe (4)
- Very safe (5)

**Risk Perception**

- During the PAST TWO WEEKS, how worried have you been about:
  - ...being infected?
    - Not at all
    - Slightly
    - Moderately
    - Very
    - Extremely
  - … friends or family being infected?
    - Not at all
    - Slightly
    - Moderately
    - Very
    - Extremely
